# Durability of Antibody Responses to SARS-CoV-2 Infection and Its Relationship to Disease Severity Assessed Using a Commercially Available Assay

**DOI:** 10.3389/fmicb.2021.770727

**Published:** 2021-12-03

**Authors:** Alanoud Alshami, Rabab Al Attas, Hadeel Anan, Aroub Al Maghrabi, Salim Ghandorah, Amani Mohammed, Abdulbary Alhalimi, Jumana Al-Jishi, Hadi Alqahtani

**Affiliations:** ^1^Department of Epidemiology and Biostatistics, King Fahad Specialist Hospital-Dammam, Dammam, Saudi Arabia; ^2^Division of Immunology, Department of Pathology and Laboratory Medicine, King Fahad Specialist Hospital-Dammam, Dammam, Saudi Arabia; ^3^Alfaisal University, Riyadh, Saudi Arabia; ^4^King Faisal University, Al-Ahsa, Saudi Arabia; ^5^Division of Infectious Disease, Department of Internal Medicine, Qatif Central Hospital, Qatif, Saudi Arabia; ^6^Division of Infectious Disease, Department of Pediatrics, King Fahad Specialist Hospital-Dammam, Dammam, Saudi Arabia

**Keywords:** SARS-CoV-2, immune response, antibody, commercial assay, disease severity, durability

## Abstract

**Background:** Assessing the humoral immune response to SARS-CoV-2 is crucial for inferring protective immunity from reinfection and for assessing vaccine efficacy. Data regarding the durability and sustainability of SARS-CoV-2 antibodies are conflicting. In this study, we aimed to determine the seroconversion rate of SARS-CoV-2 infection in a cohort of reverse-transcriptase polymerase chain reaction (RT–PCR)-confirmed SARS-CoV-2 infections and the antibody dynamics, durability, and the correlation of antibody titers with disease severity using the commercially available SARS-CoV-2 anti-spike (S1/S2) protein.

**Methods:** A total of 342 subjects with PCR-confirmed COVID-19 were enrolled. A total of 395 samples were collected at different time points (0–204) after the onset of symptoms or from the day of positive PCR in asymptomatic patients. Demographics, clinical presentation and the date of PCR were collected. All samples were tested using the automated commercial chemiluminescent system (DiaSorin SARS-CoV-2 S1/S2 IgG) on the LIAISONXL^®^ platform (LIAISON).

**Results:** The seroconversion rate for samples collected 14 days after the onset of infection was much higher than that for samples collected before 14 days (79.4% vs. 39.4%). The rate of seroconversion in symptomatic participants (62.1%) was similar to that of asymptomatic participants (56.1%) (*p* = 0.496). The IgG titer distribution was also similar across both groups (*p* = 0.142), with a median IgG level of 27.86 AU/ml (3.8–85.5) and 15 AU/ml (3.8–58.85) in symptomatic and asymptomatic participants, respectively. However, IgG titers were significantly higher in ICU patients, with a median of 104 AU/ml (3.8–179) compared to 34 AU/ml (3.8–70) in the non-ICU participants (*p* < 0.0001). Furthermore, the median time to seroconversion occurred significantly faster in ICU patients than in non-ICU participants (19 versus 47 days) (*P* < 0.0001). IgG titers were also higher in subjects ≥50 years compared to those <50 years (*p* < 0.009), male compared to female (*p* < 0.054) and non-Saudi compared to Saudi (*p* < 0.003). Approximately 74% of all samples tested beyond 120 days were positive.

**Conclusion:** Antibodies can persist in circulation for longer than 4 months after COVID-19 infection. The majority of patients with COVID-19 mounted humoral immune responses to SARS-CoV-2 infection that strongly correlated with disease severity, older age and male gender. However, the population of individuals who tested negative should be further evaluated.

## Introduction

Coronavirus disease 2019 (COVID-19) is caused by severe acute respiratory coronavirus 2 (SARS-CoV-2) and emerged in Wuhan City in December 2019. As of February 7, 2021, more than 106,000,000 cases and 2,000,000 deaths have been reported worldwide. Among those, 370,000 cases and more than 6,000 deaths have been reported in Saudi Arabia.

Coronavirus disease 2019 presents across a wide clinical spectrum ranging from asymptomatic cases to those with severe disease requiring intensive care admission (ICU) in 5% of cases (Alshami et al., 2020; Wu and McGoogan, 2020).

Currently, a new phase of the pandemic has started in which assessing the humoral immune response to SARS-CoV-2 is crucial for inferring protective immunity from reinfection and for assessing vaccine efficacy. This important knowledge will guide health policymakers when deciding who needs to receive vaccination and when. Therefore, an increasing demand exists for the development of widely commercially available valid serological assays that can infer protection from reinfection. IgG antibodies are the most abundant type in the blood postinfection and play a major role in long-term SARS-CoV-2 infection memory development and, if maintained at sufficient levels, can cause long-lasting immunity (Crotty, 2021). For most viral infections that produce immunological memory, protective immunity from reinfection lasts for years, and if infection does occur, it presents with mild symptoms (Crotty, 2021). However, this process has not been well established in SARS-CoV-2 infection. The utility of serological tests in the diagnosis and prediction of immunity against SARS-CoV-2 infection has been a topic of debate. Over time, we began to realize that the humoral immune response elicited by this virus has the same pattern of all known similar viruses, such as MERS and SARS-CoV, with a >90% seroconversion rate a few weeks post infection (Gudbjartsson et al., 2020). Many studies have reported that IgG and IgM seroconversion occurs at 10 days and peaks 49 days after the onset of symptoms (Guo et al., 2020), with a median seroconversion time between 12 and 19 days (Gudbjartsson et al., 2020; Wajnberg et al., 2020).

Knowledge regarding the characteristics of humoral immune responses to SARS-CoV-2 infection has significantly evolved over the last year; however, data regarding the durability and sustainability of SARS-CoV-2 antibodies have been conflicting (Ibarrondo et al., 2020; Self et al., 2020; Wajnberg et al., 2020).

In this study, our aim was to assess the seroconversion rate of SARS-CoV-2 infection in a cohort of participants with reverse-transcriptase polymerase chain reaction (RT–PCR)-confirmed SARS-CoV-2 infection and to examine the antibody dynamics and durability, as well as the correlation of antibody titers with disease severity using commercially available SARS-CoV-2 anti-spike (S1/S2) protein.

## Materials and Methods

This was a cross-sectional study with longitudinal follow-up of 53 subjects. This study was approved by the Central Institutional Review Board of the Ministry of Health-Saudi Arabia. Written informed consent was obtained from all recruited subjects.

### Sample Source and Time of Collection

A total of 395 serum samples were collected from 342 PCR-confirmed COVID-19 patients between May and October 2020. Samples were taken at different time points (0–204 days) after the onset of symptoms or after the date of the first positive PCR result in symptomatic and asymptomatic participants, respectively. One hundred twenty-seven (32.2%) samples were collected between 0 and 14 days, 135 (34.2%) between 15 and 59 days, 51 (12.9%) between 60 and 89 days, 51 (12.9%) between 90 and 120 days, and 31 (7.8%) at >120 days.

Out of the 342 patients, two repeat samples were obtained from 46 participants, while three repeat samples were obtained from only seven participants. Recruited participants presented with a wide spectrum of COVID-19 symptoms, ranging from asymptomatic to severely symptomatic requiring ICU admission. Patients were recruited from one quarantine facility and from COVID-19 designated hospitals in the Eastern province. In addition, some participants were recruited from a database used in a previously published article (Alshami et al., 2020). Demographic data, clinical presentation, and symptom onset were obtained from phone-based surveys for quarantined subjects and from hospital medical records for hospitalized patients. PCR data were collected from the Ministry of Health (MOH) HESN database.

We included 44 ICU patients, 257 non-hospitalized participants with mild to moderate disease not requiring oxygen or hospitalization, and 41 completely asymptomatic patients.

### Assay Performance Characteristics (Specificity, Interferences, and Potential Cross Reactivity)

All samples were tested using an automated commercial chemiluminescent system (DiaSorin SARS-CoV-2 S1/S2 IgG) on the LIAISONXL^®^ platform (LIAISON).

To assess potential cross-reactivity, we examined 40 defined samples of individuals infected with endemic coronaviruses and other respiratory viral diseases.

To assess specificity, we selected 200 prepandemic negative control samples.

Interferences were examined by testing 20 known positive samples for antinuclear antibodies (ANAs) and rheumatoid factors. No single positive sample for CoV-2 S1/S2 IgG was detected in any defined prepandemic samples, and no interference or cross reactivity was reported against potential interference substances or viral/bacterial antibodies.

Samples were stored at −80°C and tested within 1 day in batches according to the manufacturer’s instructions. None of the samples underwent more than two freeze-thaw cycles prior to analysis.

### Chemiluminescent Immunoassays

The LIAISON SARS-CoV-2 S1/S2 IgG [DiaSorin, Saluggia (VC), Italy] was used for *in vitro* quantitative detection of SARS-CoV-2 IgG in human serum. Measurements were performed on a LIAISON-XL analyzer according to the manufacturer’s instructions. The test result is shown as arbitrary units per ml (AU/ml). According to the manufacturer’s instructions, a result <12.0 is considered negative, ≥12.0 to <15.0 is considered borderline, and ≥15.0 is considered positive (Tré-Hardy et al., 2020). This test has been approved by the Food and Drug Administration (FDA) and has a reported sensitivity of 97.6% at >15 days, a specificity of 99.3%, and a positive predicted value (PPV) of 87.5%. Because all of our enrolled subjects were SARS-CoV-2 positive, we considered a result of ≥12 AU/ml as a positive test.

### Statistical Analysis

Continuous variables with skewed distributions are expressed as medians and interquartile ranges (IQRs) and were compared using the Mann–Whitney *U*-test. Normally distributed data are expressed as the mean ± standard deviation (SD). Categorical variables are expressed as numbers (%) and were compared using Fisher’s exact test. Binary logistic regression was used to predict high IgG titers that correlated with neutralizing assays. A *P*-value of <0.05 was considered statistically significant. Statistical analyses were performed using GraphPad Prism 9.

## Results

### Participants Demographics and Clinical Characteristics

Of the 342 COVID-19 patients enrolled, 57.5% (198/342) were male, and 23.4% (80/342) were non-Saudi. The mean subject age was 39.17 (±14.88). Among the 342 patients enrolled, 301 (88%) were symptomatic, 44 patients (14.6%) of whom were critically ill and required ICU admission. Two hundred fifty-seven patients were mildly or moderately symptomatic but did not require oxygen supplementation or hospital admission, and 41 were asymptomatic (Table 1).

**TABLE 1:** Baseline demographics and IgG titers stratified by disease severity.

	Asymptomatic	Symptomatic	ICU	Death
Gender (M, %)	18 (43.9%)	147 (57.2%)	33 (75%)	11 (78.6%)
Nationality (Saudi, %)	31 (75.6%)	203 (79%)	29 (66%)	10 (71.4%)
Age (y ± SD)	34.71 ± 15.8	37.53 ± 13.47	52.95 ± 14.42	52.95 ± 14.61
Median IgG titters AU/ml (IQR)	15 (3.8–58.85)	24.7 (3.8–78.65)	104 (3.8–179.3)	83.15 (3.8–103)
Total number	41	257	44	14

The clinical symptoms and demographics were significantly different between ICU and non-ICU participants. Myalgia (57.6%), cough (56%), loss of taste and smell, and headache (50.2%) were the most common symptoms observed in non-ICU participants. In contrast, cough (81.8%), dyspnea (75%), and fever (65.9%) were the most common symptoms in ICU patients (Table 2).

**TABLE 2:** Demographics and clinical characteristics of COVID-19 patients stratified by ICU status.

Symptoms	ICU	Non-ICU	*P*-value
Fever	29/44(65.9%)	106/257(41.2%)	<0.001
Dyspnea	33/44(75%)	96/257(34.2%)	<0.001
Myalgia	11/44(25%)	148/257(57.6%)	<0.001
Loss of taste or smell	4/44(9.1%)	136/257(52.9%)	<0.001
Headache	3/44(6.8%)	129/257(50.2%)	<0.001
Cough	36/44(81.8%)	144/257(56%)	0.863
Gender (M)	33/44(75%)	165/298(55.4%)	0.047
Age (χ ± SD)-y	53 ± 14.61	37.14 ± 13.82	<0.001
Nationality (Saudi, %)	29/262(11.1%)	15/80(18.8%)	0.086

Death occurred in 31.8% (14/44) of those who were admitted to the ICU. Fifty percent of those who died were ≥50 years, compared to 24.7% among those who survived (*p* = 0.055).

### SARS-CoV-2 Seroconversion

**FIGURE 1:**
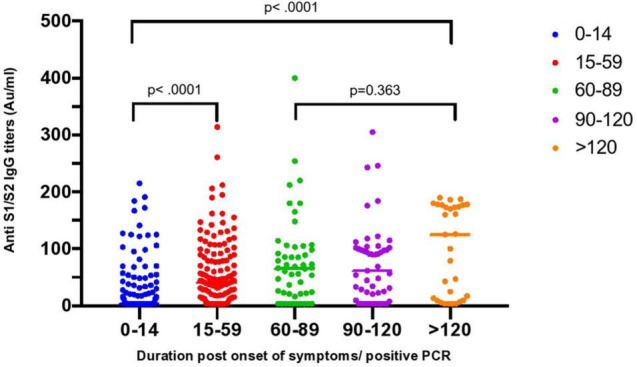
Distribution of SARS-CoV-2 anti S1/S2 protein IgG titers overtime. Grouped scatter plot showing significantly higher anti S1/S2 IgG titers in samples collected >14–59 days compared to samples collected ≤14 days post symptoms or positive PCR (*p* < 0.0001). The difference was also maintained in samples taken >120 days compared to ≤14 to ≤14 days (*p* < 0.0001). No significant difference in titers of samples collected between 60 and 89 days.

**FIGURE 2:**
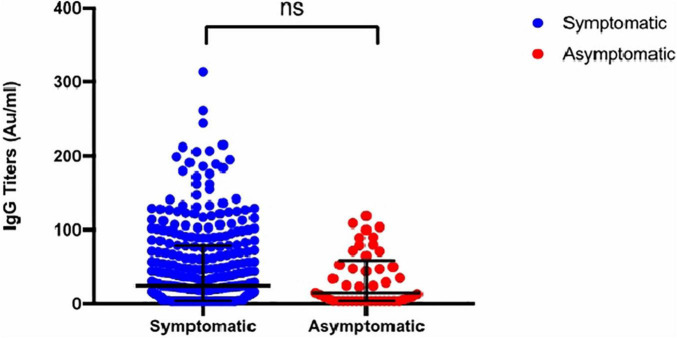
Distribution of Anti S1/S2 IgG titers stratified by symptoms. Grouped scatterplot showing no significant difference in anti S1/S2 IgG titers between symptomatic and asymptomatic participants with COVID-19 (*p* = ns).

**FIGURE 3:**
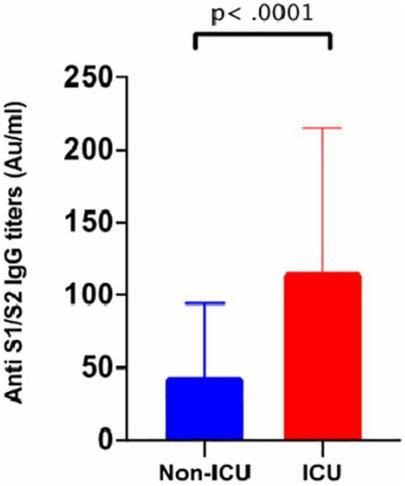
Anti S1/S2 IgG titers stratified by ICU admission. Bar chart showing that participants who required ICU admission generated significantly higher Anti S1/S2 IgG titers compared to those who did not (*p* < 0.0001).

The overall seroconversion rate for the entire cohort was 65.2% (223/342). However, the seroconversion rate for subjects whose samples were collected before 14 days was significantly lower than that for samples collected after 14 days (39.4% vs. 79.4%) (Figure 1). The median time to seroconversion was 43 days [95% confidence interval (CI): 33.50–50.96]. The rate of seroconversion in symptomatic participants (62.1%) was similar (56.1%; 23/41) to that in asymptomatic participants (*p* = 0.496). The IgG titer distribution was also similar between both groups (*p* = 0.1424), with median IgG levels of 27.86 AU/ml (3.8–85.5) and 15 AU/ml (3.8–58.85) in symptomatic and asymptomatic participants, respectively (Figure 2 and Table 1). However, a trend toward statistical significance for IgG titers ≥80 AU/ml in symptomatic compared to asymptomatic patients (*p* = 0.054) was found (Table 3). Moreover, IgG titers were significantly higher in ICU patients, with a median of 104 AU/ml (3.8–179) compared to 34 AU/ml (3.8–70) in the non-ICU participants (*p* < 0.0001), as shown in Figure 3. Furthermore, the median time to seroconversion occurred significantly more rapidly in ICU patients than in non-ICU patients (19 versus 47 days) (*p* < 0.0001).

**TABLE 3:** Multivariant logistic regression analysis for predictors of high IgG antibody titers after SARS-CoV-2 infection.

Dependent variable	Independent variables	Coefficient	Adjusted odds ratio	*P*-value	95% confidence interval
IgG titers ≥80*Au*/*ml*	ICU admission	1.607	4.526	*P* < 0.0001	2.138–9.583
	Age ≥50	0.772	2.165	*P* = 0.009	1.208–3.879
	Non-Saudi	0.872	2.392	*P* = 0.003	1.336–4.823
	Gender (M)	0.66	1.934	*P* = 0.052	0.993–3.768
IgG titers ≥120*Au*/*ml*	ICU admission	1.88	6.556	*P* < 0.0001	2.972–14.464
	Age ≥50	0.84	2.322	*P* = 0.024	1.118–4.823
	Non-Saudi	0.856	2.355	*P* = 0.022	1.129–4.912
	Gender	0.887	2.429	*P* = 0.033	1.076–5.485

### Seroconversion Rate of Participants With Two Serology Samples

**FIGURE 4:**
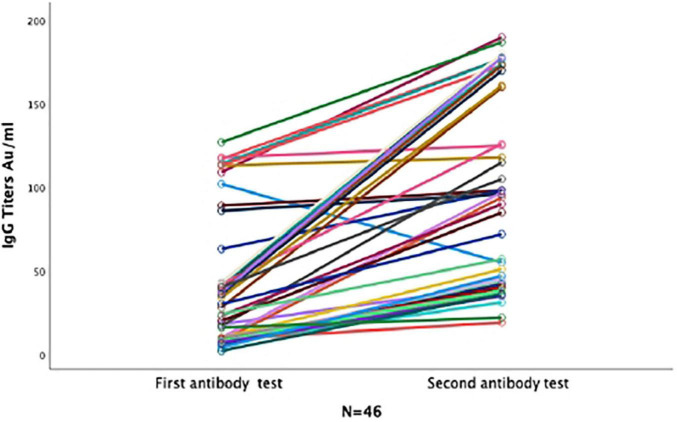
Anti S1/S2 IgG dynamics in forty-six participants with two repeated serological samples. Line graph for 46 participants with two serological tests. Each line represents one participant. All participants who seroconverted in the first test remained positive in the second test. Majority of participants who tested negative in the first sample seroconverted in the second sample.

Of the 46 patients who had a repeat test, seroconversion occurred in 11 (23.9%) patients who were previously negative, 52.2% (24/46) continued to have positive titers at a median of 92 days (40.75–163.25), nine (19.6%) continued to be negative, and two (4.3%) lost their antibodies at 72 and 178 days post-symptom onset and postpositive PCR, respectively (Figure 4). Six of nine participants who did not seroconvert on the second test were female, with a median age of 31 years (19.5–47.5), and the median time from symptom onset to testing was 42 days (33.5–64.5).

### Seroconversion Rate of Participants With Three Serology Samples

**FIGURE 5:**
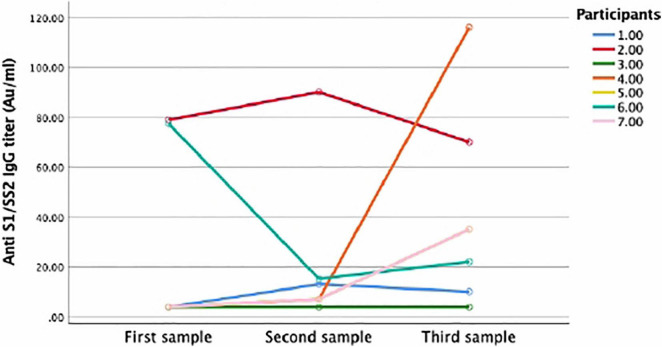
Anti S1/S2 IgC dynamics for participants with three repeated samples. Line graph showing the dynamics of anti S1/S2 IgG overtime. Seven participants had three repeated samples overtime. Each line represents one participants. Five participants tested positive in the third sample (2, 3, 4, 5, and 7). One participant lost his antibodies (1) and one did not seroconvert in all samples (6).

Of the seven patients who had a third sample at a median follow-up time of 102 (79–127), five were positive, of whom one was still positive at 204 days. At that time, two had seroconverted, three remained positive, one remained negative, and one had lost his antibodies (Figure 5).

One hundred twenty-seven samples were collected between 0 and 14 days, out of which 75 (59%) were obtained at <10 days, and only 28% of them were positive. The median IgG level was 4 AU/ml (3.8–35.9), as shown in Table 4.

**TABLE 4:** Number of serological assays performed across different time points and their median IgG levels.

Duration (days)	Number of samples	Number of positive samples	Median IgG (IQR)	Sensitivity (95% CI)
0–14	127	50	4(3.8−35.9)	39.4% (0.815–1.085)
15–59	135	111	41.4(15−89.3)	82.2% (0.879–1.022)
60–89	51	41	66(21−103)	78.8% (0.824–1.075)
90–120	51	38	61.6(9.5−102)	74.5% (0.811–1.088)
>120	31	23	125(9.56−177)	74.2% (0.766–1.134)
Total	395	263		

The median times between the first, second, and third serology tests and positive RT–PCR results were 18 (8–64.25), 92 (40.75–163.25), and 94 days (74–107), respectively.

### Predictors of High Antibody Titers

Using binary logistic regression, we found that ICU admission increased the odds of developing high IgG titers by 4.89 compared to non-ICU participants (*P* < 0.001). Furthermore, non-Saudi participants were associated with a 2.39-fold greater chance of developing high IgG titers than Saudi patients (*p* = 0.003). Finally, age >50 years at the time of infection was associated with a 2.3-fold greater odds of developing high titers than age <50 years (*p* = 0.009) (Table 3).

### Change of Assay Sensitivity and Antibody Titers Over Time

The sensitivity of DiaSorin’s assay in detecting SARS-CoV-2 infection antibodies varied over time. During the first 14 days, the sensitivity was only 39.4% (50/127); however, the sensitivity significantly increased to 82.2% (111/135) in serum samples tested between 15 and 59 days compared to between 0 and 14 days (*p* < 0.0001), 78.8% (41/52) between 60 and 89 days, and then dropped again to 74.5% (23/32) in samples tested between 90 and 120 days and 74.2% in samples tested >120 days from symptom onset or from the day of positive PCR in asymptomatic patients (Table 4).

The anti-SARS-CoV-2 IgG level distribution was significantly different across the four duration categories (*p* < 0.001), from which the highest IgG levels were observed in samples collected at >120 days, followed by samples taken between 60 and 89 days, during which the lowest titers were observed in the first 2 weeks (Figure 1). Thirty-one samples from 29 subjects, of whom two (6.5%) were ICU patients, nine (29%) were >50 years, and 19 (61.3%) were male, were collected after 120 days.

## Discussion

Although we are more than 1 year into the COVID-19 pandemic, our understanding of the components of protective immune responses to SARS-CoV-2 infection remains limited. Although emerging evidence suggests that both components of the adaptive immune response, including T and B cells, play a major role in controlling SARS-CoV-2 infection, (Chen and John Wherry, 2020; Sekine et al., 2020) more emphasis has been placed on antibody detection because antibodies are easiest to measure.

Neutralizing antibodies (NtAbs) that measure the functional capabilities of host antibodies to neutralize and kill the virus are a hot research topic. Several studies have reported a strong correlation between in-house neutralizing assays and protection from reinfection. Nevertheless, few studies have examined the correlation between neutralizing activities measured using neutralizing assays and commercially available anti-S protein IgG assays. These studies have shown a strong correlation between antibodies against SARS-CoV-2 S protein and NtAb50 titers. Among the commercially available serological assays tested, the LIAISON^®^ SARS-CoV-2 S1/S2 IgG (DiaSorin) assay outperformed other assays in predicting NtAb50 titers of 1/160 against SARS-CoV-2 with reported accuracies of >92, 98, and 95% at 80, 90, and 124 AU/ml, respectively (Bonelli et al., 2020; Legros et al., 2021; Valdivia et al., 2021). Furthermore, Weidner et al. (2020) reported that low antibody titers detected using LIAISON^®^ SARS-CoV-2 S1/S2 IgG (<30 AU/ml) exhibited a strong negative predictive value (97.3%) for NtAb. This multiple reported correlation between DiaSorin assay and NtAb has directed our choice of using the DiaSorin assay as a proxy for NtAb50. However, none of these assays were able to predict the trajectory of NtAbs over time; in fact, some studies have suggested a lack of correlation (Oved et al., 2020). Importantly, the seroconversion rate differs significantly according to the timing of sample collection, serological assay performance characteristics, the tested SARS-CoV-2 target proteins, and perhaps based on some intrinsic differences in individual immune responses (Valdivia et al., 2020).

Characterization of humoral immune responses to SARS-CoV-2 infection is important for the prediction of protection from reinfection and for testing the efficacy of vaccination. Neutralization assays that test the capability of host serum to prevent a live virus from infecting cell cultures are believed to be a surrogate indicator for SARS-CoV-2 protection and prevention of reinfection (Addetia et al., 2020). However, these tests are impractical to deploy on a large scale because they require a high-containment laboratory with biosafety cabinets. Thus, serological assays that correlate well with neutralization assays are needed for use in large-scale epidemiological studies.

In our study, we tested 342 subjects with confirmed SARS-CoV-2 infection based on RT–PCR who presented a wide range of symptoms (41 asymptomatic, 257 mild to moderate non-hospitalized, and 44 critically ill ICU patients) using the LIAISON^®^ SARS-CoV-2 S1/S2 IgG commercial assay to assess the overall seroconversion rate, antibody kinetics, variability, and durability of antibody titers in relation to the severity of symptoms and subjects’ demographics.

The overall seroconversion rate of SARS-CoV-2 infection in our cohort (65.2%) was lower than previously reported results. However, this rate is similar to studies in which the DiaSorin assay was used. This similarity could be due to multiple reasons: (1) the sensitivity of commercially available assays with respect to detecting IgG anti-spike protein antibodies increases over time, most likely due to SARS-CoV-2 S protein antibody dynamics (Bao et al., 2021). Importantly, in our study, approximately 1/3 (127) of the samples were collected between 0 and 14 days after symptom onset or after obtaining a positive PCR test result, of which 75 samples were collected after ≤10 days, resulting in a sensitivity of 39.4% during this period. However, the sensitivity significantly increased over time to reach 82.2% (*p* < 0.00001) in samples collected between 15 and 59 days and then dropped slightly to 71% for samples collected after 120 days (Table 2). Our findings are similar to studies that used DiaSorin assays with reported sensitivities of 10–25% in samples <7 days and 80–90% in samples taken after 14 days with overall sensitivity ranging between 50 and 83.1% and a specificity of 98% (Long et al., 2020; Weidner et al., 2020; Mahase, 2021).

(2) The second explanation for the low seroconversion rate in our study could be the use of a single serological assay that targeted only the spike protein. Combining serological assays that target different SARS-CoV-2 proteins (nucleocapsid and spike) has been shown to improve sensitivity (Turbett et al., 2021). (3) The third explanation could be that this observed negative seroconversion could truly indicate negative seroconversion. Although this finding cannot be inferred from sera collected before 14 days after symptom onset or a positive PCR result, eight of 53 patients (15.1%) with repeated samples remained negative at a median follow-up time of 92 days (40.75–163). Although it is difficult to draw conclusions based on the small sample size and based on a test that only targets the spike protein domain, those patients were all mildly symptomatic and were not different from the seroconverts in terms of their demographics and clinical presentation (Table 1). Only a few studies have reported cases of primary non-converters after 14 days based on a positive PCR (Marklund et al., 2020). In a study performed by Oved et al. (2020) it was found that ∼ 6.4% of their cohort was non-seroconverted after being tested by three different serological assays. Lumley et al. (2021) reported a group of patients who were confirmed to be COVID-19 positive based on RT–PCR but were seronegative, and those seronegative patients were prone to SARS-CoV-2 reinfection. Another report by Zhang et al. observed a 51-year-old female with persistent negative antibodies 14 days after infection (Schwartz et al., 2020). If we believe that SARS-CoV-2 antibodies are the major players in postinfection protective immunity, our findings indicate that a subgroup of the population with negative seroconversion would likely be at higher risk for reinfection, and screening patients based on serological assays might underestimate the prevalence of COVID-19. Currently, >106,000,000 cases of COVID-19 have been reported worldwide, with very few anecdotal reports of reinfection. We have evidence from non-human studies that SARS-CoV-2 infection prevents reinfection in rhesus macaques after re-exposure to the virus (Chandrashekar et al., 2020). The largest human study that infers postinfection protection is the SIREN UK public health trial. In this trial, ∼20,000 health care workers were enrolled, of which 6,660 had positive SARS-CoV-2 infection based on PCR tests and positive serology tests. Over a 5-month follow-up, only 44 out of the positive patients developed reinfection, inferring an 83% protection rate from future reinfection (Mahase, 2021). Furthermore, Long et al. (2020) demonstrated that patients with asymptomatic infection have lower IgG titers than symptomatic patients, and 40% of asymptomatic patients lose their titer over a short period of time. Sekine et al. (2020) demonstrated robust T memory cells after mild and asymptomatic COVID-19 infection in the absence of antibodies. This study again supports the key role of memory T cells in the development of immune protection. It also demonstrates that the concept of waning antibodies from the circulation after viral infection is a normal aspect of the immune system and does not necessarily correlate with loss of protective immunity. Out of the 53 repeated samples, only three (5.7%) participants lost their antibodies.

The median anti-spike protein IgG titers were similar among asymptomatic and symptomatic patients, excluding ICU patients (*p* = 0.239). Nevertheless, participants with more severe disease, in particular ICU patients, exhibited significantly higher median antibody titers than non-ICU patients (*p* < 0.00001). These findings have been observed in many studies (Hashem et al., 2020; Piccoli et al., 2020). Overall, these results indicate that only those with severe disease can elicit robust humoral immune responses and NtAbs; however, the role of these strong antibodies in slowing disease activity and severity has yet to be determined.

To further predict the ability of subjects to produce antibodies that correlate with NtAbs, we used the same two cutoff levels (80 and 120 AU/ml) of anti-spike protein IgG antibodies measured by the DiaSorin assay, which was validated as a surrogate marker for predicting NtAb activity with high accuracy, as done in previous studies.

We found that a history of ICU admission was the strongest predictor for producing high antibody titers for both cutoff points. Interestingly, non-Saudis individuals exhibited an increased risk for producing higher postinfection titers [adjusted odds ratio (OR): 2.33, 95% CI = 1.336–4.823]. This finding may indicate that non-Saudi participants present with a more severe course than Saudi participants, although this observation did not reach statistical significance in our study (Table 1, *p* = 0.085). Although only a trend toward significance in our study for racial disparity in COVID-19 was observed, a large systematic review involving 50 studies from the United States and United Kingdom concluded that individuals of black and Asian ethnicities have a higher risk of contracting COVID-19 in general, and more specifically, Asians were at a higher risk of hospital admission and death (Sze et al., 2020).

Moreover, male sex was also associated with increasing odds of producing higher antibody titers only when titers ≥120 AU/ml were used (adjusted OR: 2.43, CI = 1.076–5.485), and it was associated with an increased risk of ICU admission (*p* = 0.047).

Our study is the largest to assess the dynamics, durability and predictors of humoral immune responses in Saudi Arabia for COVID-19. Unlike most studies that measure the humoral immune response in hospitalized patients, approximately 80% of the enrolled subjects were either asymptomatic or mildly symptomatic, which is closer to the real epidemiology of COVID-19.

Our study is not without limitations. First, we used a single commercial test that targeted one specific protein (spike protein) to assess the overall seroconversion rate; this specificity could have underestimated the overall seroconversion rate and could cause antibody misclassification bias. Iyer et al. (2020) demonstrated that adding another isotype, such as anti-IgA and/or IgM, to samples obtained within the first 14 days postinfection yielded a slight increase (9%) in the test’s accuracy. Second, the number of subjects who had longitudinal follow-up samples was limited in our study, which affected our ability to assess individual variability in antibody kinetics; however, the wide range of testing at different time points allowed us to draw solid conclusions regarding the durability of the antibodies. Last, antibody neutralization was not performed and was inferred based on previous studies that identified a strong correlation between the DiaSorin and neutralization results.

## Conclusion

The majority of patients with COVID-19 mounted humoral immune responses to SARS-CoV-2 infection that persisted for 4 months and strongly correlated with disease severity, older age and male sex. However, a subgroup of participants who tested negative existed, which needs further evaluation in future studies.

## Data Availability Statement

The original contributions presented in the study are included in the article/supplementary material, further inquiries can be directed to the corresponding author/s.

## Ethics Statement

The studies involving human participants were reviewed and approved by the Central Institutional Review Board of Saudi Arabia. The patients/participants provided their written informed consent to participate in this study.

## Author Contributions

AlA and RA: study conception and design. ArA, RA, HAn, AbA, JA-J, and AM: data collection. AlA: data analysis. AAM, RA, HAn, AM, and HAl: manuscript writing and revision. All authors contributed to the article and approved the submitted version.

## Conflict of Interest

The authors declare that the research was conducted in the absence of any commercial or financial relationships that could be construed as a potential conflict of interest.

## Publisher’s Note

All claims expressed in this article are solely those of the authors and do not necessarily represent those of their affiliated organizations, or those of the publisher, the editors and the reviewers. Any product that may be evaluated in this article, or claim that may be made by its manufacturer, is not guaranteed or endorsed by the publisher.
